# Common Cold Coronavirus Test Positivity Decreased After Widespread SARS-CoV-2 Experience

**DOI:** 10.1093/ofid/ofaf326

**Published:** 2025-06-18

**Authors:** Trisha Parayil, Janet Monroe, David J Bean, Manish Sagar

**Affiliations:** Department of Medicine, Boston University Chobanian & Avedisian School of Medicine, Boston, Massachusetts, USA; Department of Medicine, Beth Israel Medical Center, Boston, Massachusetts, USA; Department of Virology, Immunology and Microbiology, Boston University Chobanian & Avedisian School of Medicine, Boston, Massachusetts, USA; Department of Medicine, Boston University Chobanian & Avedisian School of Medicine, Boston, Massachusetts, USA; Department of Virology, Immunology and Microbiology, Boston University Chobanian & Avedisian School of Medicine, Boston, Massachusetts, USA

**Keywords:** heterotypic immunity, influenza, RSV, SARS-CoV-2, seasonal coronaviruses

## Abstract

**Background:**

Diverse respiratory viruses, such as the common cold coronaviruses (ccCoVs), respiratory syncytial virus (RSV), and influenza virus (IV), circulated before the emergence of severe acute respiratory syndrome coronavirus 2 (SARS-CoV-2), the causative agent of coronavirus disease 2019 (COVID-19). We hypothesized that ccCoV, but not RSV or IV, test positivity changed after widespread SARS-CoV-2 infection and COVID-19 vaccination because of shared features among the coronaviruses (CoVs).

**Methods:**

We collected all ccCOVs, RSV, and IV detected at Boston Medical Center from October 2015 to April 2024. We compared virus positivity in the 5 respiratory seasons before the emergence of SARS-CoV-2 in March 2020 with the 2 seasons after the SARS-CoV-2 Omicron variant surge ended around April 2022. We used multivariate linear regression, generalized estimating equations, and multivariate logistic regression analysis to compare total weekly virus detected and test positivity frequency.

**Results:**

Test positivity for ccCoVs, but not RSV or IV, was significantly lower after widespread SARS-CoV-2 infection and COVID-19 vaccination compared with the seasons before the first documented SARS-CoV-2 case. There was ∼60% lower odds of a ccCoV, but no change in RSV odds, in the seasons after extensive established SARS-CoV-2 immunity from infection and COVID-19 vaccination.

**Conclusions:**

Our results suggest that ccCoV, but not RSV and IV, positivity has decreased significantly in recent respiratory seasons. There are multiple potential mechanisms for the observed ccCoVs decrease, such as cross-reactive immune response among the different but highly related CoVs, along with changing behavioral and health care practices.

Even before the emergence of severe acute respiratory syndrome coronavirus 2 (SARS-CoV-2), the causative agent of coronavirus infectious disease 2019 (COVID-19), virus respiratory infections were common, especially in the fall and winter seasons. Most viral respiratory infections caused by different microbiologic pathogens have indistinguishable clinical signs and symptoms [1, 2]. Furthermore, health care providers often do not conduct microbiologic tests to identify specific viral pathogens, primarily because there are limited therapeutics and most mild respiratory infections resolve without intervention. Thus, a specific microbiologic diagnosis, based on clinical criteria and laboratory testing, often remains unknown.

In the pre-COVID-19 seasons, influenza virus (IV), respiratory syncytial virus (RSV), and common cold coronaviruses (ccCoVs) were some of the most commonly detected viral pathogens among tested individuals presenting with a respiratory infection [3, 4]. Unlike IV and RSV, shared genetic and structural similarity provides heterotypic immunity among the ccCoVs, such as human coronavirus (HCoV)-OC43, HCoV-HKU-1, HCoV-NL63, HCoV-229E, and SARS-CoV-2 [5, 6]. All coronaviruses (CoVs) have similar genome architecture, and some gene segments share >60% similarity [7]. Multiple investigations demonstrated that individuals sampled before the COVID-19 pandemic harbored preexisting immune responses against SARS-CoV-2, and this immunity likely emerged from prior ccCoV infections [8, 9]. Furthermore, prior documented ccCoV infection associates with lower COVID-19 severity, but there is no difference in SARS-CoV-2 infection incidence [10]. Recently, we also demonstrated that in individuals with rigorously classified antigen exposure history, previous SARS-CoV-2 infection, but not COVID-19 vaccination, associates with decreased ccCoV disease [11]. We conducted this investigation before the emergence of highly infectious SARS-CoV-2 Omicron variants, which led to widespread infections even in the presence of preexisting COVID-19 vaccination [12]. After the SARS-CoV-2 Omicron variant surge ebbed around April 2022 in Boston, it has become difficult to confidently ascertain infection history because individuals often test at home or omit diagnostic testing entirely. Furthermore, by April 2022 >80% of the individuals in Boston had completed the recommended primary COVID-19 vaccination series [13, 14]. Here, we examined the change in disease incidence at a population level caused by ccCoVs after nearly ubiquitous SARS-CoV-2 Omicron infections and COVID-19 vaccinations. We compared ccCoVs with RSV and IV because these pathogens share seasonality, transmission modes, and disease signs and symptoms [1].

## METHODS

### Study Design and Data Collection

This single-center retrospective observational study at Boston Medical Center (BMC) compared ccCoV, RSV, and IV disease incidence before the COVID-19 pandemic and after the SARS-CoV-2 Omicron surge and widespread COVID-19 vaccination. We collected all ccCoV (HCoV-OC43, HCoV-HKU-1, HCoV-NL63, and HCoV-229E), RSV, and IV genomic or antigen-based molecular test results at BMC from April 30, 2015, to September 1, 2024. Over the study period, health care providers used the comprehensive respiratory panel polymerase chain reaction (CRP-PCR; Biofire Diagnostics), respiratory mini panel PCR (BioMerieux), Cobas-liat (Roche), and Cobas point of care (Roche) to detect respiratory pathogens. All of these tests have reported sensitivity and specificity of >95% [15, 16]. All testing decisions were at the discretion of the health care provider or based on BMC clinical algorithms. No patient contributed >1 test result on the same day. A positive test with multiple ccCoVs or a mixture of ccCoVs, RSV, or IV counted once each for the different outcomes of interest.

### Statistics

All years were divided into 52 weeks, and the leap years contained different numbers of days for week 9 (February 26 to March 4). We defined peak respiratory season as week 40 (starting October 1) to the next year week 13 (until April 1). Thus, full respiratory seasons contained 26 weeks. The last pre-COVID-19 respiratory season ended on March 11, 2020, because non-SARS-CoV-2 respiratory infections were low following the start of the COVID-19 pandemic on March 11, 2020 [1, 3, 17]. In Boston, the SARS-CoV-2 Omicron surge began around December 2021 and abated around April 2022 [18, 19]. We designated the 5 pre-COVID-19 (10/1/2015–03/11/2020) and the 2 post–widespread Omicron infection and COVID-19 vaccination (10/1/2022–4/1/2024) seasons as Period 1 and Period 2, respectively.

We used generalized estimating equations (GEE) to compare corresponding weekly totals in peak respiratory seasons in Period 1 and Period 2. In the GEE population-averaged model, the number of weekly infections or tests was the dependent variable and the classified period was the predictor, with the specific week as a grouping variable in an exchangeable correlation. We also used an alternative time-series linear regression model. In this model, the number of weekly infections and the classified period were the dependent and independent variables, respectively. Each specific week during a respiratory season was a categorical dummy variable. We used multivariate logistic regression analysis to predict the odds of a positive test in the 2 different periods. The test result was the dependent variable, and the period, categorically defined age, biological sex at birth, and level of hospitalization (outpatient or hospital setting) were the dependent variables. The regression and logistic regression analyses did not account for an individual's SARS-CoV-2 infection, COVID-19 vaccination, or other antigen exposure history because we did not have access to this information at a population level.

In our previous reports, we observed a ∼50% reduction in ccCoV detection in those with prior documented SARS-CoV-2 infection [11]. Thus, we assumed that there would be a ∼50% lower ccCoV detection in Period 2 as compared with Period 1. In Period 1, there was an average (range, SD) of 14.35 (0–48, 11.43) ccCoVs detected per week during a respiratory season [3]. We estimated that we would have 90% power to detect a statistically significant 50% difference (2-sided *P* value of .05, 2-sample means test) by comparing a minimum of 52 weeks each between Period 1 and Period 2. Furthermore, an average (range, SD) of 5.85% (0%–22.9%, 4.77%) of the all ccCoV weekly tests were positive in Period 1 [3]. We estimated that there would be 80% power to detect a statistically significant 50% decrease (2-sided *P* value of .05, 2-sample means test) by assessing a minimum of 44 weeks each between the 2 periods.

All *P* values represent 2-sided tests. We performed statistical analyses with Stata, version 17 (StataCorp).

### Patient Consent

The Boston University Institutional Review Board approved the data collection without obtaining written consent from the individuals.

## RESULTS

**Figure 1.**
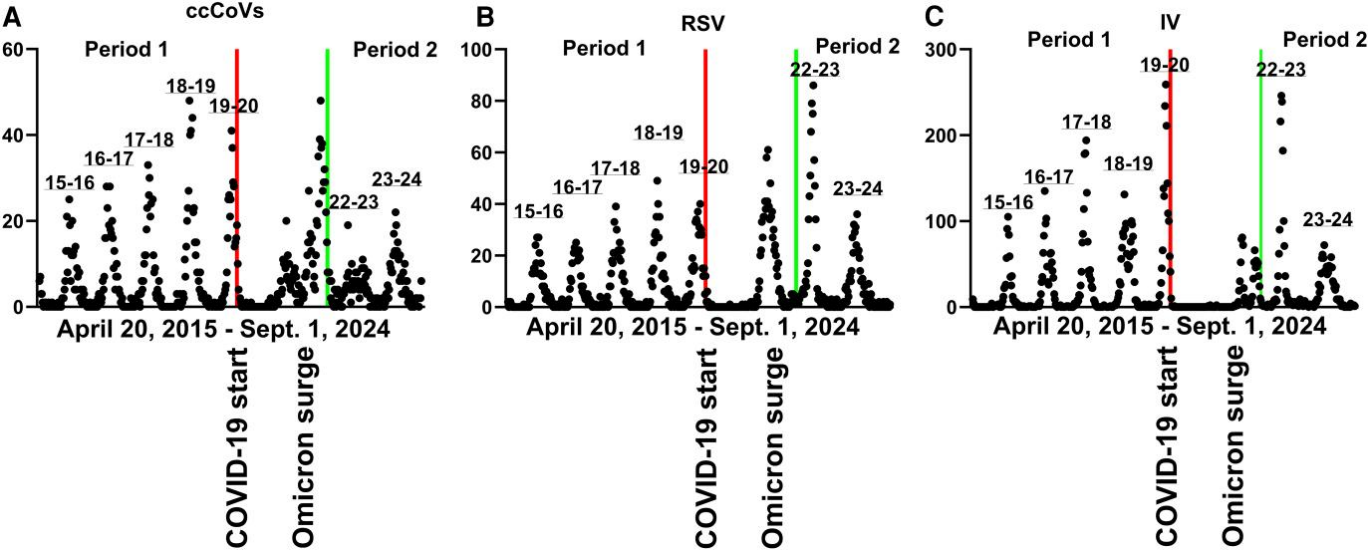
Respiratory virus detection at Boston Medical Center from April 2015 to September 2024. Weekly detected ccCoV (*A*), RSV (*B* ), and IV (*C* ) from April 20, 2015, to September 30, 2024 (x-axis). Each individual dot represents a weekly total. The years on top identify the respiratory seasons. In each graph the left and right solid line indicate the start of the COVID-19 pandemic and the time after the SARS-CoV-2 Omicron surge period respectively. Note the y-axes have different scales. Abbreviations: ccCoV, common cold coronavirus; COVID-19, coronavirus disease 2019; IV, influenza virus; RSV, respiratory syncytial virus; SARS-CoV-2, severe acute respiratory syndrome coronavirus 2.

The CRP-PCR comprised all ccCoVs tests (85 661) and diagnoses (3121). Similarly, 97.11% of the RSV tests (88 543) and 98.38% of the detected cases (4076) used CRP-PCR. IV testing varied, but the CRP-PCR comprised 65.67% of all IV tests (130 963) and 54.62% of all positives (9586). The ccCoV (Figure 1*A*), RSV (Figure 1*B*), and IV (Figure 1*C*) infections followed a seasonal pattern, with peaks between week 40 and subsequent year week 13 before the COVID-19 pandemic (2015–2020), termed Period 1, and the years after the Omicron surge and widespread COVID-19 vaccination (2022–2024) [20, 21], classified as Period 2.

**Figure 2.**
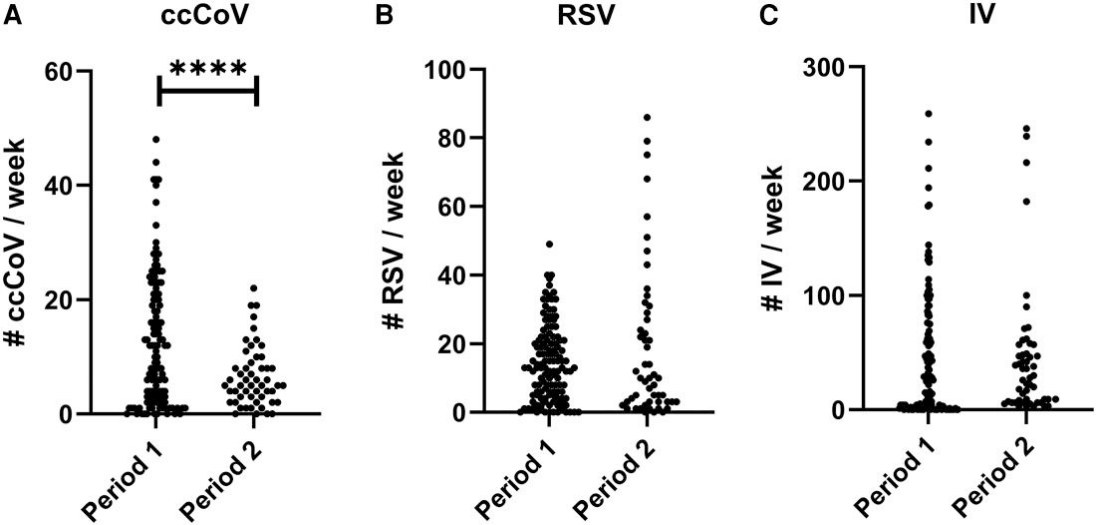
Respiratory virus detection in the 2 different periods. Each dot represents the weekly total number of detected ccCoV (*A*), RSV (*B* ), and IV (*C* ) during the different periods. Period 1 encompasses 5 seasons before the COVID-19 pandemic. Period 2 incorporates 2 seasons after widespread SARS-CoV-2 Omicron variant infection and near universal COVID-19 vaccination. ****P value <.0001 from a generalized estimating equations model. Abbreviations: ccCoV, common cold coronavirus; COVID-19, coronavirus disease 2019; IV, influenza virus; RSV, respiratory syncytial virus; SARS-CoV-2, severe acute respiratory syndrome coronavirus 2.

We compared weekly totals during the peak respiratory seasons in 5 prepandemic Period 1 years with 2 Period 2 years after widespread SARS-CoV-2 antigen exposure from infection and vaccination. Weekly ccCoVs were ∼50% lower in Period 2 as compared with Period 1 (intercept, 12.35. β = –5.87; 95% CI, −8.01 to −3.73; *P* < .0001) (Figure 2*A*). On the other hand, RSV (intercept, 14.55; β = 4.34; 95% CI, –0.44 to 9.12; *P* = .08) (Figure 2*B*) was not lower in Period 2. In addition, the total numbers of tests that could detect ccCoVs (*P* = .93) (Supplementary Figure 1*A*) and RSV (*P* = .76) ( Supplementary Figure 1*B*) were not significantly different in the 2 periods. Health care providers conducted nearly 2-fold more tests that detected IV (intercept, 220.04; β = 220.80; 95% CI, 186.25 to 255.35; *P* < .0001) (Supplementary Figure 1*C*), but IV weekly totals were not significantly higher in Period 2 as compared with Period 1 (intercept, 41.86; β = 2.83; 95% CI, –13.46 to 19.12; *P* = .73) (Figure 2*C*), even with the greater testing. Similarly, the Centers for Disease Control and Prevention characterized the 2 Period 2 respiratory seasons as “moderate” severity, similar to most of the 5 Period 1 prepandemic years [22].

We also used a linear regression time-series model to confirm these findings. In these models, ccCoV (Table 1), but not RSV (Supplementary Table 1) or IV (Supplementary Table 2), detection was significantly lower in Period 2 as compared with Period 1. This model confirmed that the majority of detected infections occurred during the winter weeks. Thus, longer peak respiratory seasons defined as beginning before week 40 and ending in the subsequent year after week 14 yielded similar results. The ccCoV linear regression showed a coefficient of determination of 0.65, suggesting that the model robustly predicted the proportion of observed variance. Low numbers of HCoV-OC43, HCoV-HKU-1, HCoV-NL63, and HCoV-229E precluded examining incidence differences in specific ccCoVs in the different periods.

**Table 1.** ccCoV Period 1 vs Period 2 and Weekly Changes

Variable	Estimate (β)	95% CI	*P* Value
Constant	2.22	−2.81 to 7.25	Reference
Period 2	−5.78	−7.95 to −3.60	<.001
Peak respiratory season weeks			
10/01–10/07	Reference		
10/08–10/14	0.86	−6.20 to 7.92	.81
10/15–10/21	0.86	−6.20 to 7.92	.81
10/22–10/28	0.00	−7.06 to 7.06	1.00
10/29–11/04	1.14	−5.92 to 8.20	.75
11/05–11/11	1.57	−5.49 to 8.63	.66
11/12–11/18	1.86	−5.20 to 8.92	.60
11/19–11/25	3.14	−3.92 to 10.20	.38
11/26–12/02	5.57	−1.49 to 12.63	.12
12/03–12/09	7.14	0.08 to 14.20	.05
12/10–12/16	8.86	1.80 to 15.92	.01
12/17–12/23	12.43	5.37 to 19.49	<.001
12/24–12/31	17.29	10.22 to 24.35	<.001
01/01–01/07	19.29	12.22 to 26.35	<.001
01/08–01/14	20.00	12.94 to 27.06	<.001
01/15–01/21	20.14	13.08 to 27.20	<.001
01/22–01/28	24.71	17.65 to 31.78	<.001
01/29–02/04	21.86	14.80 to 28.92	<.001
02/05–02/11	20.57	13.51 to 27.63	<.001
02/12–02/18	18.57	11.51 to 25.63	<.001
02/19–02/25	14.14	7.08 to 21.20	<.001
02/26–03/04	11.14	4.08 to 18.20	<.001
03/05–03/11	10.86	3.80 to 17.92	<.001
03/12–03/18	5.70	−1.65 to 13.05	.13
03/19–03/25	7.20	−0.15 to 14.55	.06
03/26–04/01	6.04	−1.31 to 13.39	.11

Abbreviation: ccCoV, common cold coronavirus.

**Figure 3.**
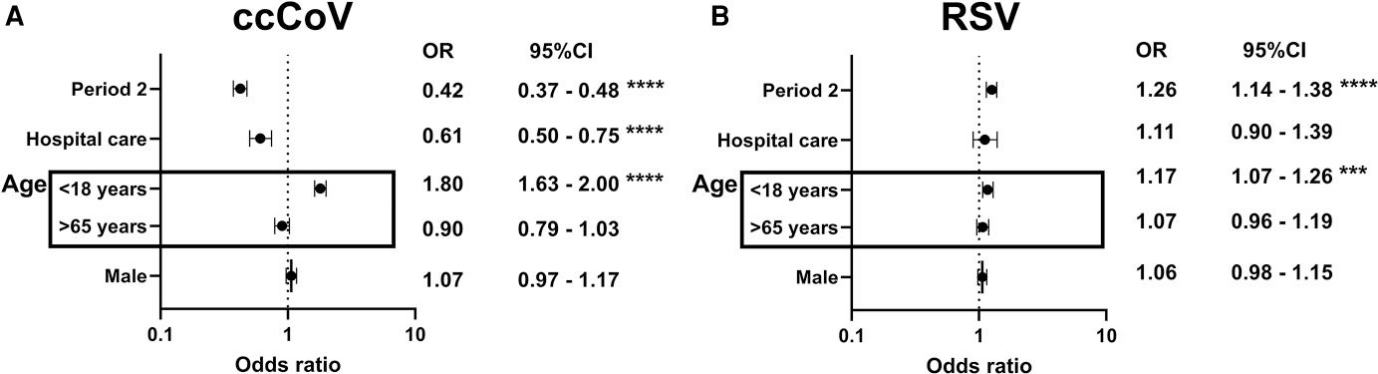
Test positivity in the 2 different periods. Adjusted OR for ccCoV (*A*) and RSV (*B*) detection that accounts for age, level of medical care, and biological sex at birth. For each variable, OR (x-axis) associated with the presence of the factor (y-axis), and the 95% CI is shown. The dotted line indicates equivalent odds (OR, 1) of disease. ***P < .001; ****P < .0001. Abbreviations: ccCoV, common cold coronavirus; OR, odds ratio; RSV, respiratory syncytial virus.

We conducted multivariate logistic regression analysis to account for differences in patient demographics and level of medical care. There were >50% lower odds of detecting a ccCoV in Period 2 as compared with Period 1 after accounting for age, gender, and level of medical care (Figure 3*A*). On the other hand, there was around 25% greater odds of detecting RSV in Period 2 (Figure 3*B*). CcCoV, but not RSV, disease had lower association with hospitalization as compared with outpatient care. Individuals age <18, but not >65, years had significantly greater odds of ccCoV and RSV disease incidence [23, 24]. Biological sex at birth did not associate with ccCoV or RSV disease. We did not conduct multivariable logistic regression analysis for IV because the positivity rate would change with the nearly 2-fold greater testing in the post-Omicron years.

## DISCUSSION

Investigations have not examined changes in respiratory disease incidence after widespread SARS-CoV-2 Omicron infections and near universal COVID-19 vaccination. Immediately following the start of the COVID-19 pandemic, there was a generalized decrease in all respiratory virus transmission. This occurred due to non-virus-specific mechanisms, such as physical distancing, school closures, masking, and potentially a general antiviral environment at the initial site of infection [1, 3, 17]. It was unlikely that there was a virus-specific immune response responsible for the documented decline. In contrast, we observed that ccCoV disease incidence significantly decreased in the recent respiratory infection seasons compared with the pre-COVID-19 years. There was, however, no significant change in RSV or IV. It is possible, although unlikely, that changes in patient behavior, health care or hospital access, and physician testing and treatment approaches yielded a ccCoV-specific decline. In general, patients do not know the identity of the infecting virus to influence changes in masking frequency, social gathering, and medical care–seeking behavior. Furthermore, health care providers generally cannot distinguish these viruses based on clinical grounds alone because ccCoV, RSV, and IV have similar signs and symptoms [1, 2]. Therefore, this would not change the likelihood of diagnostic testing or other clinical decisions in the absence of knowledge about the specific infecting virus. Thus, we postulate that CoV-specific heterotypic immunity elicited from prior SARS-CoV-2 antigen exposure associates with reduced ccCoV-induced disease.

It is possible that disease severity differs among ccCoV, RSV, and IV. Diagnostic testing may be pursued only in those with severe disease, and this could influence both the likelihood that patients present for clinical evaluation and health care provider behavior. The significantly increased IV testing in Period 2 as compared with Period 1 may reflect that health care providers pursued diagnostic evaluation primarily for those with more symptomatic disease, such as an especially virulent influenza strain. In Period 2, however, there was “moderate” influenza activity, similar to the pre-COVID seasons [22]. On the other hand, there may have been greater IV testing because of the availability of bundled SARS-CoV-2 and IV diagnostics and the available therapies for these 2 infections. Thus, health care providers may have more readily pursued IV testing. Our observations are from test results on patients who presented for clinical care, and thus we characterized virus detection as representing disease rather than asymptomatic infection. Post-Omicron, more ccCoV infections may be asymptomatic, not meriting a clinical visit, and thus the total number of ccCoV infections may remain unchanged [5]. Thus, changes in disease severity may influence the hospital patient volume and test positivity observed for different respiratory viruses.

In our previous study, we observed no change in SARS-CoV-2 infection incidence among those with as compared with those without a prior documented ccCoV infection [10]. Individuals with a recent ccCoV infection, however, had subsequent milder COVID-19. We have also demonstrated that individuals with prior SARS-CoV-2 infection, but not COVID-19 vaccination, had decreased likelihood of subsequent ccCoV disease [11]. Postinfection immune responses, which influence disease outcome, and not neutralizing antibodies, which provide sterilizing protection, associated with decreased ccCoV disease incidence and COVID-19 severity [9, 11, 25]. In addition, we observed circulation of all 4 ccCoVs after the Omicron surge (Supplementary Figure 2), and this is different from the observed extinction of IV Yamagata strain [17]. In aggregate, this suggests that a prior CoV infection ameliorates subsequent disease severity from a heterologous CoV, but it does not protect against infection.

This study has limitations because it shows associations and does not prove causation. Furthermore, generalizability will require observations from other health centers. Our current work demonstrates changes in ccCoV epidemiology in the city of Boston after nearly ubiquitous exposure to SARS-CoV-2 antigens from infection and COVID-19 vaccination. Although our prior study suggests that SARS-CoV-2 infection potentially provides this heterotypic immunity [11], we cannot discount the effect of COVID-19 vaccination in this investigation. Ongoing CoV evolution and waning SARS-CoV-2 immunity may modify this association in the future.

## Supplementary Material

ofaf326_Supplementary_Data

## Supplementary Data

Supplementary materials are available at *Open Forum Infectious Diseases* online. Consisting of data provided by the authors to benefit the reader, the posted materials are not copyedited and are the sole responsibility of the authors, so questions or comments should be addressed to the corresponding author.
