# Supplementary material for: Common Cold Coronavirus Test Positivity Decreased After Widespread SARS-CoV-2 Experience
Source: Open Forum Infect Dis. 2025 Jun 18;12(7):ofaf326. doi: 10.1093/ofid/ofaf326 (PMC12207968; doi:10.1093/ofid/ofaf326)
Supplement: ofaf326_Supplementary_Data [file ofaf326_supplementary_data.zip › Supplementary Figure Captions OFID_R2.docx]

**Supplementary Figure 1.** **Respiratory virus tests in the two periods.** Each dot represents a weekly total of ccCoV (A), RSV (B), and IV (C) tests during the peak respiratory seasons in Period 1 and Period 2. **** indicates p – value < 0.0001, and it is from a generalized estimating equations model.

**Supplementary Figure 2, Virus detection from May 2015 to September 2024 at Boston Medical Center.** Weekly detected HCoV-OC43 (black), HCoV-HKU-1 (red), HCoV-NL63 (blue), and HCoV-229E (green) from April 30, 2015 to September 30, 2024. Each individual dot represents weekly total. The dotted lines denote the periods of interest.
