# Supplementary material for: Common Cold Coronavirus Test Positivity Decreased After Widespread SARS-CoV-2 Experience
Source: Open Forum Infect Dis. 2025 Jun 18;12(7):ofaf326. doi: 10.1093/ofid/ofaf326 (PMC12207968; doi:10.1093/ofid/ofaf326)
Supplement: ofaf326_Supplementary_Data [file ofaf326_supplementary_data.zip › Supplementary Tables.docx]

**Supplementary Table 1. RSV Period 1 versus Period 2 and weekly changes.**

| **Variable** | **Estimate (β)** | **95% CI** | **p-value** |
| --- | --- | --- | --- |
| **constant** | 7.56 | -3.64 – 18.75 | REFERENCE |
| **Period 2** | 4.54 | -0.30 – 9.38 | 0.07 |
| **PEAK RESPIRATORY SEASON WEEKS** | | | |
| **10/01 - 10/07** | REFERENCE | | |
| **10/08 - 10/14** | 0.86 | -14.85 – 16.57 | 0.91 |
| **10/15 - 10/21** | 4.29 | -11.43 – 20.00 | 0.59 |
| **10/22 - 10/28** | 7.29 | -8.43 – 23.00 | 0.36 |
| **10/29 - 11/04** | 7.43 | -8.28 – 23.14 | 0.35 |
| **11/05 - 11/11** | 13.71 | -2.00 – 29.43 | 0.09 |
| **11/12 - 11/18** | 12.00 | -3.71 – 27.71 | 0.13 |
| **11/19 - 11/25** | 10.00 | -5.71 – 25.71 | 0.21 |
| **11/26 - 12/02** | 9.29 | -6.43 – 25.00 | 0.25 |
| **12/03 - 12/09** | 12.57 | -3.14 – 28.28 | 0.12 |
| **12/10 - 12/16** | 13.43 | -2.28 – 29.14 | 0.09 |
| **12/17 - 12/23** | 13.86 | -1.85 – 19.57 | 0.08 |
| **12/24 - 12/31** | 15.14 | -0.57 – 30.85 | 0.06 |
| **01/01 - 01/07** | 17.14 | 1.43 – 32.85 | 0.03 |
| **01/08 - 01/14** | 10.86 | -4.85 – 26.57 | 0.17 |
| **01/15 - 01/21** | 10.57 | -5.14 – 26.28 | 0.19 |
| **01/22 - 01/28** | 11.29 | -4.43 – 27.00 | 0.16 |
| **01/29 - 02/04** | 10.00 | -5.71 – 25.71 | 0.21 |
| **02/05 - 02/11** | 7.57 | -8.14 – 23.28 | 0.34 |
| **02/12 - 02/18** | 2.57 | -13.14 – 18.28 | 0.75 |
| **02/19 - 02/25** | 1.43 | -14.28 – 17.14 | 0.86 |
| **02/26 - 03/04** | 0.71 | -15.00 – 16.43 | 0.93 |
| **03/05 - 03/11** | -3.57 | -19.28 – 12.14 | 0.65 |
| **03/12 - 03/18** | -2.24 | -18.59 – 14.11 | 0.79 |
| **03/19 - 03/25** | -3.74 | -20.09 – 12.61 | 0.65 |
| **03/26 – 04/01** | -6.07 | -22.43 – 10.28 | 0.46 |

**Supplementary Table 2. Influenza Period 1 versus Period 2 and weekly changes.**

| **Variable** | **Estimate (β)** | **95% CI** | **p-value** |
| --- | --- | --- | --- |
| **constant** | 1.06 | -37.29 – 39.41 | REFERENCE |
| **Period 2** | 2.79 | -13.79 – 19.38 | 0.33 |
| **PEAK RESPIRATORY SEASON WEEKS** | | | |
| **10/01 - 10/07** | REFERENCE | | |
| **10/08 - 10/14** | 0.29 | -53.54 – 54.11 | 0.99 |
| **10/15 - 10/21** | 0.71 | -53.11 – 54.54 | 0.98 |
| **10/22 - 10/28** | 0.86 | -52.96 – 54.68 | 0.98 |
| **10/29 - 11/04** | 3.43 | -50.39 – 57.25 | 0.90 |
| **11/05 - 11/11** | 7.71 | -46.11 – 61.54 | 0.78 |
| **11/12 - 11/18** | 11.14 | -42.68 – 64.96 | 0.68 |
| **11/19 - 11/25** | 16.43 | -37.39 – 70.25 | 0.55 |
| **11/26 - 12/02** | 38.00 | -15.82 – 91.82 | 0.17 |
| **12/03 - 12/09** | 47.14 | -6.68 – 100.96 | 0.09 |
| **12/10 - 12/16** | 50.14 | -3.68 – 103.96 | 0.07 |
| **12/17 - 12/23** | 42.43 | -11.39 – 96.25 | 0.12 |
| **12/24 - 12/31** | 41.57 | -12.25 – 95.39 | 0.13 |
| **01/01 - 01/07** | 41.57 | -12.25 – 95.39 | 0.13 |
| **01/08 - 01/14** | 56.14 | 2.32 – 109.96 | 0.04 |
| **01/15 - 01/21** | 54.86 | 1.04 – 108.68 | 0.05 |
| **01/22 - 01/28** | 84.00 | 30.18 – 137.82 | <0.001 |
| **01/29 - 02/04** | 93.71 | 39.89 – 147.54 | <0.001 |
| **02/05 - 02/11** | 98.29 | 44.46 – 152.11 | <0.001 |
| **02/12 - 02/18** | 80.00 | 26.18 – 133.82 | <0.001 |
| **02/19 - 02/25** | 65.57 | 11.75 – 119.39 | 0.02 |
| **02/26 - 03/04** | 53.14 | -0.68 – 106.96 | 0.05 |
| **03/05 - 03/11** | 43.86 | -9.96 – 97.68 | 0.11 |
| **03/12 - 03/18** | 41.84 | -14.18 – 97.87 | 0.14 |
| **03/19 - 03/25** | 44.68 | -11.35 – 100.70 | 0.12 |
| **03/26 – 04/01** | 44.34 | -11.68 – 100.37 | 0.12 |
