# Supplementary figures and images for: Common Cold Coronavirus Test Positivity Decreased After Widespread SARS-CoV-2 Experience
Source: Open Forum Infect Dis. 2025 Jun 18;12(7):ofaf326. doi: 10.1093/ofid/ofaf326 (PMC12207968; doi:10.1093/ofid/ofaf326)

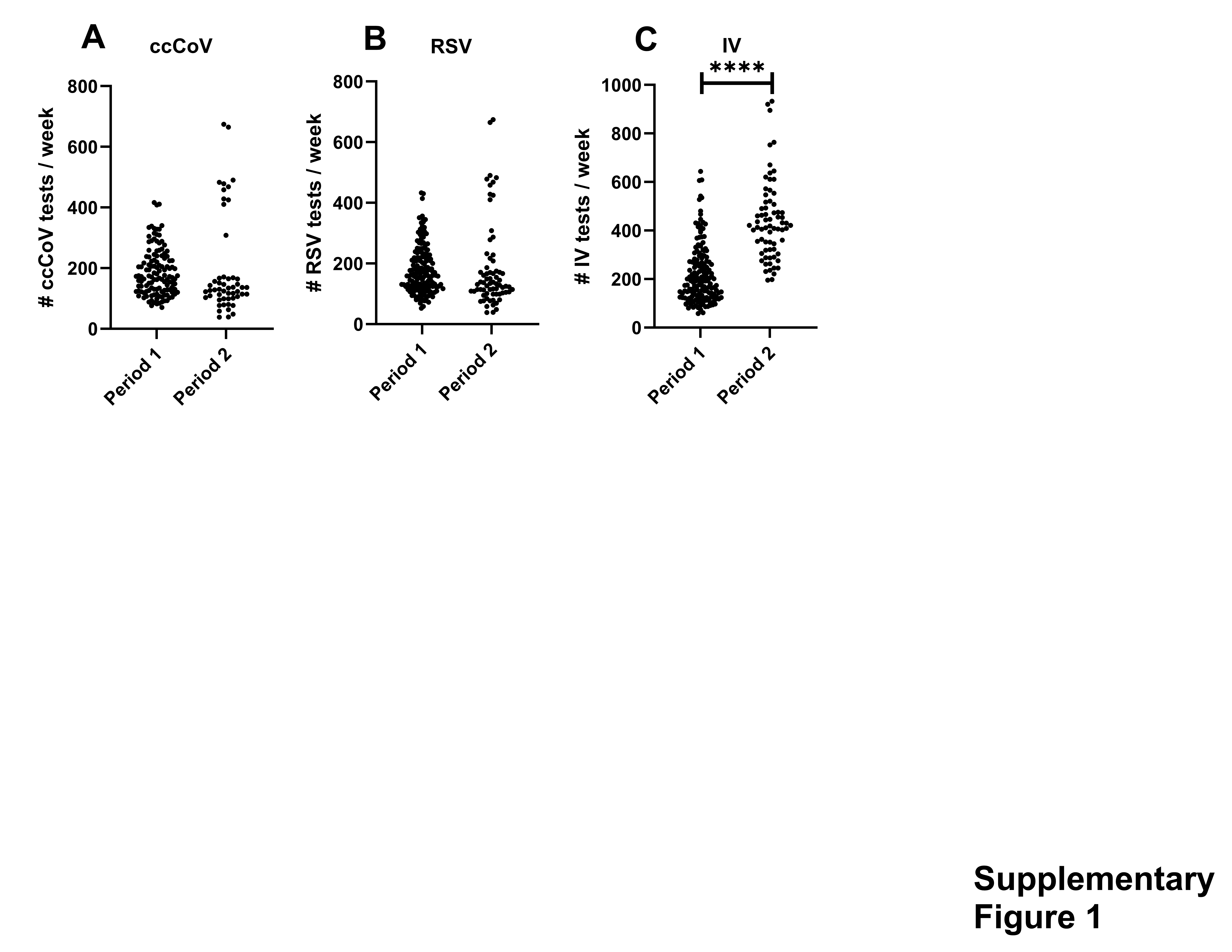

Supplement: ofaf326_Supplementary_Data [file ofaf326_supplementary_data.zip › Supplementary Figure 1.tif]

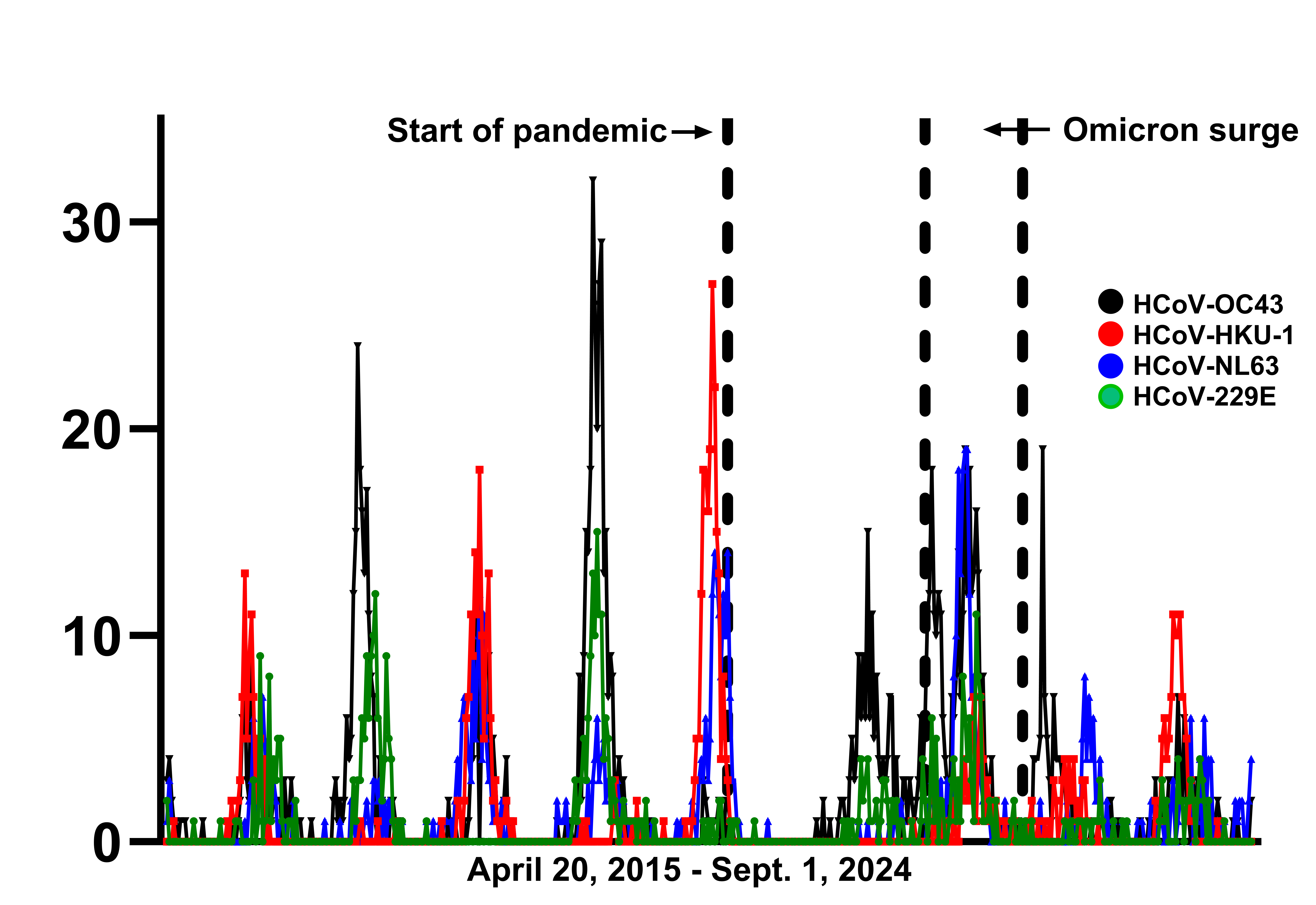

Supplement: ofaf326_Supplementary_Data [file ofaf326_supplementary_data.zip › Supplementary Figure 2.tif]
